# Face Mask Use and Social Distancing Attitude of Healthcare Students: A Multi-Disciplinary Study

**DOI:** 10.3390/healthcare11060901

**Published:** 2023-03-21

**Authors:** Jonas Preposi Cruz, Ejercito Mangawa Balay-Odao, Junel Bryan Bajet, Jennifer Mesde, Khalaf Alotaibi, Ahmed Almogairi, Nahed Alquwez, Mohammed Alqahtani, Ahmed Mansour Almansour, Sharifa Alasiry, Jazi Shaydied Alotaibi

**Affiliations:** 1 Department of Medicine, School of Medicine, Nazarbayev University, Astana 010000, Kazakhstan; 2 School of Advanced Studies, Saint Louis University, Baguio 2600, Philippines; 3 Department of Nursing, College of Applied Medical Sciences, Shaqra University, Al-Dawadmi 11961, Saudi Arabia; 4 College of Applied Medical Sciences, Department of Nursing, King Faisal University, Alahsa 31982, Saudi Arabia; 5 Department of Nursing, College of Applied Medical Sciences, Majmaah University, Al-Majmaah 11952, Saudi Arabia

**Keywords:** COVID-19, face mask use, medicine, nursing, pharmacy, social distancing

## Abstract

This multi-disciplinary, cross-sectional, and descriptive study investigated health care students’ face mask use knowledge, attitude, and practices (KAP) and social distancing attitudes. The study was carried out from October to December 2021 and surveyed 543 health care students selected via convenience sampling from the three Shaqra University campuses in Saudi Arabia. Standard multiple linear regressions were conducted on face mask use KAP and social distancing attitude to identify their significant associated factors. The students in this study had poor knowledge and a neutral attitude towards and modest use practices of face masks during the COVID-19 pandemic. Being female, being a nursing student, and having greater self-reported COVID-19-prevention knowledge were related to higher levels of face mask use knowledge. Having higher face mask use knowledge was linked to better attitude and practice. Knowledge of COVID-19 and its prevention and decreased COVID-19 risk perception were associated with better face mask use practices. The students had more positive than negative attitudes toward social distancing. Having known someone who was infected by the virus, lower risk perception, and better face mask use practices were associated with more positive social distancing attitudes. The findings imply the need to ensure that future health care practitioners are knowledgeable, have a positive attitude and good practices concerning face mask use, and have positive attitudes toward social distancing. The study implications are relevant for health care education in Saudi Arabia and elsewhere.

## 1. Introduction

The COVID-19 cases reported by many countries are continuously increasing, although the fatality rate remains low [1,2]. COVID-19 transmission is airborne from infected individuals [2,3,4]. In addition, a person may become infected when touching their eyes, nose, or mouth after touching surfaces or objects that the virus has contaminated [4]. Face mask (FM) use and social distancing help the government and health authorities control the present pandemic’s spread [1]. Lednicky et al. [5]. stated that the recommended social distancing is beyond two meters. However, low-quality FMs may not provide good protection from COVID-19. The study done by Pushpawela et al. [6]. found significant variability in the particle penetration and the pressure drop of face masks. It tested multiple N95, KN95, procedure, and cloth masks. The study found consistent performance only of N95 masks, and the degree of protection provided by other types of masks was uncertain.

FM use and social distancing [3] are effective in mitigating the transmission of the virus, but the attitudes of people from different countries vary [4]. Additionally, FM use and social distancing have significantly reduced mental problems such as fear and anxiety during this pandemic [3].

Given the evidence that these preventive measures are effective [5], public compliance is very important However, studies have shown barriers to compliance with social distancing [7] and FM use [8]. These are related to work requirements, mental and physical wellbeing issues, the sufficiency of other measures such as handwashing, and the overreaction of the community to COVID-19 [5]. A person’s compliance with preventive health measures depends on their knowledge, attitude, and practices (KAP) [7]. As mentioned by Martinelli et al., individuals who are knowledgeable about their risk of COVID-19 are likely to practice wearing an FM and social distancing [9].

To help enhance preventive measures against future infectious disease outbreaks, this study investigated KAP on FM use and social distancing attitudes among health care students. Health authorities and universities can apply this to implementing policy for adherence to these measures. The mask use KAP [6] and social distancing attitude [10] of health care students play a vital role in understanding their acceptance and compliance. However, the actual KAP on FM use and social distancing attitude of health care students remain unknown in the Kingdom of Saudi Arabia (KSA). Thus, this study may guide intervention development on these measures. This work also helps address conflict toward FM use and social distancing acceptance of health care students during a pandemic.

### Background

SARS-CoV-2 is a severe virus strain that spreads rapidly and has caused a health burden worldwide [11]. As the number of infections escalates in the KSA, the government and health authorities have implemented measures to prevent and control the transmission of the virus [12,13]. These have included lockdown, quarantine, restriction of international and local flights, and increasing public awareness of the significance of regular handwashing, social distancing, and FM use. The implementation of these measures is related to the unknown complete pathophysiology of COVID-19 and confusing information on the mode of transmission, which is believed to be by droplet and contact [1]. FM use to control the increase of respiratory disorders is simple, but the KAP in every country varies [14]. The acceptance of public FM use has been debated in some European countries [15]. On the other hand, in Vietnam, China, Japan, and Korea, implementation has been less challenging because wearing an FM is common in public places for health and cultural reasons [4]. This may explain the finding of a study in Vietnam that showed that the public had good KAP regarding FM use [8]. A study in China revealed that 98% of people used FMs and followed the mass masking policy [16]. The decision to wear an FM is associated with social and cultural practices, health-related concerns, personal meaning, and ethical and political meaning [14]. Wearing an FM is an act of altruism and self-protection [15]. This finding is also highlighted in the study of Martinelli et al., who stated that a person’s behavior toward wearing an FM is associated with social responsibility [9].

A person’s knowledge about COVID-19 affects their FM use practice [17]. The good practice of wearing an FM is observed more among health care students, such as those in nursing and pharmacy [8]. The high FM use score of pharmacy and nursing students is associated with their training in medical school and their proactive role as future medical practitioners [18]. However, a Chinese study reported no correlation between wearing FMs and educational background [11]. Duong et al. found that gender was associated with FM use [12]. This was also observed in studies in China [19] and Saudi Arabia [17].

Another measure that assisted many countries in containing the increasing number of COVID-19 cases has been strict social distancing policies [20]. Although these are beneficial, the attitude and understanding of individuals significantly affect the effectiveness of the policy implementation [21]. Thus, measuring health care students’ attitudes to and understanding of social distancing is crucial because they will be implementing the policy in the future. Limited studies have been conducted on this aspect compared with the growing body of knowledge concerning the KAP of health care students regarding COVID-19 in KSA. This study is relevant now given that a new variant of COVID-19 is emerging, and FM use and social distancing are still recommended for protection and prevention. Recently, the Ministry of the Interior reimposed FM use and social distancing in public places in KSA to prevent the spread of the new variant.

## 2. Materials and Methods

### 2.1. Aims

This study assessed health care students’ facemask use KAP and social distancing attitudes. It also examined the factors associated with these variables among health care students in Saudi Arabia.

### 2.2. Design

This multi-disciplinary study adopted a cross-sectional, descriptive, and quantitative design.

### 2.3. Participants

The study was conducted on the three campuses of Shaqra University. This university offers health care and other courses. The health care courses offered are medicine, pharmacy, and allied courses (nursing and medical laboratory specialist). The study participants were students who were officially enrolled in the college of medicine, pharmacy, nursing, and medical laboratory specialist studies at the three campuses. The participants were 18 years old and above and willing to partake in the investigation. Students who were absent when the data were collected or unwilling to join were excluded. A total of 543 students determined by convenience sampling participated in the study from October to December 2021. A priori power analysis was performed using the G*Power version 3.1 to determine the required sample size for this study. Since there were four regression models in the study, we considered the model with the highest number of predictor variables in the power analysis. Using an α-error probability of 0.05, statistical power of 80.0%, medium effect size (0.15), and 17 predictor variables, the needed sample size was 127. Therefore, the current sample size is more than adequate to detect a medium effect size at 80.0% statistical power [22].

### 2.4. Instruments

Demographic data were obtained to describe the participants. Two instruments were used in the study, with permission granted by the authors: the Coronavirus Social Distance Attitude Scale (CSDAS) [23] and the Face Mask Use Scale (FMUS) [24].

The CSDAS is a tool used to measure a person’s positive and negative attitudes about social distancing. The tool uses a 5-point Likert scale from 1 (strongly disagree) to 5 (strongly agree). It consists of fourteen items: eight items measure positive social distancing attitude, and six measure negative social distancing attitude [23]. The CSDAS has exhibited excellent internal consistency. The internal consistency of the positive social distancing attitude was 0.92, and the negative social distancing attitude was 0.91, which are both interpreted as high [23]. The overall Cronbach’s alpha for the CSDAS in our sample was 0.890. For the negative and positive social distancing attitude items, alpha coefficients of 0.837 and 0.895 were computed, respectively.

The FMUS is a tool used to measure the KAP of health practitioner students on FM use [24]. The tool consists of thirty-three items: five items for knowledge, nineteen for attitude, six for measuring the practice of FM use, and three for demographics and medical history data. The knowledge part of the tool measures proper FM use, the correct placement for FM coverage, the placement of the FM wire, and the proper positioning of the sides of the mask. It uses multiple-choice questions that require a correct answer. If the participant answers the question correctly, then a score of 1 is given; if the answer is incorrect, 0 is given. Thus, the knowledge score ranges from 0 to 5. The attitude items are rated with a 5-point Likert scale ranging from “strongly disagree” to “agree strongly”. The scale is collapsed into three categories for data analysis. If the participant answers “strongly disagree” or “disagree”, it is categorized as a negative attitude and equivalent to a −1 score. If the answer is uncertain, then the category is undecided and equivalent to a 0 score. If the answer is “agree” or “strongly agree”, it is categorized as a positive attitude and equivalent to a score of 1. Thus, the attitude score on FM use ranges from −19 to 19. Lastly, the practice item measures the use of FMs for self-protection or the protection of others at home, at the clinic, and in public places. The items are rated from “never” to “always” for data analysis. The 5-point Likert scale is collapsed into three categories. A score of 0 is given for a participant who answers “never” or “rarely” to wearing an FM, a score of 1 is given if the answer is “sometimes”, and a score of 2 is given for “always” wearing an FM. The score for practice thus ranges from 0 to 12 [24]. Chong et al. evaluated the psychometric properties of the FMUS and reported that the CVI was 100%, and the Cronbach alpha coefficient was 0.81 [25]. In our sample, the FMUS exhibited acceptable reliability. The Kuder–Richardson formula 20 coefficient for the FM use knowledge and practice items in the FMUS was 0.707 and 0.891, respectively, and the Cronbach’s alpha for the FM use attitude items was 0.920.

### 2.5. Data Collection

Upon approval of the study, the researchers sought approval to administer the questionnaires from the deans of different colleges at the three campuses. After permission was granted, the researchers administered the questionnaire to the participants, or the department head administered it if they requested this. The questionnaires were distributed during the free time of the students so as not to disrupt their studies. If participants had any concerns about the study, they were instructed to contact the primary author. The researcher checked and evaluated the questionnaires for any missing data. The data were tallied, tabulated, and analyzed. The data were secured in a folder file on the personal computer of the researcher.

### 2.6. Ethical Consideration

A letter of request and ethical approval to conduct the study was secured from the Ethics Standing Committee at Shaqra University (ERC_SU_20210038). The committee ensured the ethical conduct of the study. Confidentiality was observed by ensuring that the data were not associated with the participants. The consent form addressed the aims, purpose, and research process, and these were discussed during data gathering with the participants before they were requested to sign a consent form. During the data gathering, the participants had the right to ask questions or withdraw. Coercion was avoided by respecting the participants’ rights and voluntary participation.

### 2.7. Data Analysis

Data analyses were performed using SPSS version 22.0. Descriptive statistics (i.e., mean, standard deviation, frequency count, and percentage) were used to treat the study variables. Standard multiple linear regressions were conducted on FM use KAP and social distancing attitude to identify their significant associated factors. The demographic characteristics and COVID-19-related variables were entered as the predictor variables. Dummy variables were developed for the following predictor variables: academic level, program, and having persons in their immediate community who were infected with the virus. FM use knowledge was included as a predictor for FM use attitudes. FM use knowledge and attitudes were added as predictor variables for FM use practices. For the social distancing attitudes, FM use practice was included as a predictor variable. Before performing the regression analyses, the tests for assumptions were conducted. In all the regression models, the P-P plots showed that the residuals indicated normal distributions. Scatterplots reflected evenly dispersed residuals, implying a non-violation of the assumption of homoscedasticity. All the tolerance values were >0.20, and all the VIF values were <10, signifying a lack of violations of multicollinearity. Thus, standard multiple linear regression analyses were conducted. Before performing the regression analyses, the tests for assumptions were conducted: normal distribution of the residuals of the regression, “homoscedasticity”, “multicollinearity”, and linear relationship of the variables [26]. In all the regression model, the P-P plots showed that the residuals indicate normal distributions. Moreover, scatterplots reflect evenly dispersed residuals implying a non-violation of the assumption of homoscedasticity. All the tolerance values were >0.20 and all the VIF values were <10, signifying the lack of violations on multicollinearity. According to Lobiondo-Wood and Haber, if the test of assumptions reveals that the residuals are normally distributed and homoscedastic, the linearity assumption is also met [26]. Therefore, standard multiple linear regression analyses were appropriate to conduct [26].

## 3. Results

### 3.1. Demographic Characteristics and COVID-19-Related Information

Among the 543 health care students surveyed in this study, more than half were men (50.6%), and the sample had an average age of 22.70 years (SD = 4.70). The greatest proportion of students were in their sophomore year (32.8%), and the fewest were in their internship year (6.8%). The majority of the participants were nursing students (54.3%), followed by pharmacy students (21.7%), medicine students (14.4%), and clinical laboratory students (9.0%). Most students belonged to a nuclear family (54.3%). More than two-thirds of the students had a previous COVID-19 infection (71.3%), and more than half knew someone in their immediate social community with a confirmed case of COVID-19 (54.3%). On a scale of 1 to 10, their perceived knowledge about COVID-19 and its prevention was 6.72 (SD = 2.57) and 7.54 (SD = 2.30), respectively (Table 1). The mean score for risk perception and fear of COVID-19 was 3.09 (SD = 0.94) and 3.23 (SD = 1.09), respectively.

### 3.2. Face Mask Use Knowledge

The students’ mean knowledge score on FM use was 3.36 (SD = 1.57), with a possible score of 0 to 7. As shown in Supplementary Tables S1–S3, the majority of the students did not know how to correctly use a mask (53.0%) or know which side of the FM should face the outside (69.8%). Similarly, a majority of the students incorrectly thought that “a cloth facial mask is as effective as a regular surgical facial mask” (60.6%), and “a facial mask helps to prevent human immunodeficiency virus” (71.1%). In contrast, most of the students knew “the purpose of the metal strip on the face mask” (68.5%), “the need to cover the mouth when sneezing or coughing even with a face mask” (60.6%), and not to reuse the FM even when not sick (61.3%).

The regression model in Table 2 indicated that sex, program, previous COVID-19 infection and perceived COVID-19 knowledge were significantly related to the students’ FM use knowledge (*F*_15,527_ = 5.66, *p* < 0.001). Women had higher knowledge than men (ß = 0.81, *p* < 0.001, 95% CI = 0.50, 1.12). Pharmacy students exhibited lower knowledge compared with nursing students (ß = −0.36, *p* = 0.048, 95% CI = −0.72, 0.00). A point increase in the perceived COVID-19-prevention knowledge scores corresponded to a 0.09-unit increase in the FM use knowledge of the students (*p* = 0.014, 95% CI = 0.02, 0.16).

### 3.3. Face Mask Use Attitudes

Regarding the attitudes of the students toward FM use, the average score was 1.78 (SD = 3.99), indicating a near-neutral attitude. A summary of the findings on the attitudes of the students toward FM use is shown in Supplementary Table S4. The following factors were identified as significantly associated with the students’ attitudes toward FM use: year level, program, and FM use knowledge (*F*_16,526_ = 2.57, *p* = 0.001). Specifically, third-year students showed more positive attitudes compared with second-year students (ß = 1.00, *p* = 0.027, 95% CI = 0.11, 1.89). Students in the medicine (ß = 1.35, *p* = 0.015, 95% CI = 0.26, 2.44) and pharmacy (ß = 1.05, *p* = 0.031, 95% CI = 0.10, 1.99) programs showed better attitudes towards FM use than did students in the nursing program. A point increase in FM use knowledge corresponded to a 0.27-unit increase in FM use attitudes (*p* = 0.021, 95% CI = 0.04, 0.49; Table 3).

### 3.4. Face Mask Use Practices

Most of the participants frequently or always wore an FM when in public areas (52.5%) and when going to a doctor’s clinic (54.0%) to protect themselves from influenza-like illness (ILI). Additionally, most used an FM in public (52.5%) and in the doctor’s clinic when they had symptoms of ILI (57.5%). An almost equal proportion of students never or rarely (35.5%) and frequently or always wore an FM at home when they had ILI symptoms. More students never (27.1%) or sometimes (27.4%) wore an FM at home when a family member had ILI (Supplementary Table S5).

Table 4 shows the significant factors linked with students’ FM use practices based on the multiple regression analysis (*F*_17,525_ = 14.81, *p* < 0.001). Third-, fourth-, and internship-year students had better FM use practices than second-year students. An increase in knowledge of COVID-19 (ß = 0.31, *p* < 0.001, 95% CI = 0.17, 0.45) and its prevention (ß = 0.23, *p* = 0.005, 95% CI = 0.07, 0.38) was associated with better FM use practices. An increased COVID-19 risk perception was related to poor FM use practices (ß = −0.85, *p* < 0.001, 95% CI = −1.21, −0.49). An increased score on FM use knowledge corresponded to better FM use practices (ß = 0.28, *p* = 0.005, 95% CI = 0.08, 0.47).

### 3.5. Attitudes toward Social Distancing

The positive and negative attitudes on social distancing among the students were examined. The mean positive and negative attitude scores were 3.39 (SD = 0.96) and 2.84 (SD = 0.92), respectively, implying that the students possessed more positive attitudes towards social distancing. Supplementary Table S6 reflects the descriptive analyses of the variables related to the attitude toward social distancing among the students.

As reflected in Table 5, fourth-year and internship-year students had poorer attitudes towards social distancing than did second-year year students. Students who knew individuals with confirmed COVID-19 had more positive attitudes than those who did not know anyone infected with the virus. An increase in the risk perception score was associated with a poor social distancing attitude (ß = −0.07, *p* < 0.001, 95% CI = −0.11, −0.03). Better FM use practices were associated with a more positive social distancing attitude (ß = 0.04, *p* < 0.001, 95% CI = 0.03, 0.05).

## 4. Discussion

This study explored health care students’ FM use KAP and social distancing attitudes. The results highlight that the students had suboptimal knowledge of FM use, as evidenced by the mean score of 3.36 (SD = 1.57) out of a possible 7. Similar studies in Malaysia [6] and Ethiopia [27] reported poor knowledge among medical students of FM use. The suboptimal FM use knowledge could be related to the students’ lack of interest in exploring the proper use of FM because most of them had learned how to use personal protective equipment. This lack of interest among health care students could also be associated with the lack of interest among students to read manufacturer instructions on proper FM use and decontamination [28].

This report also reflects that most of the students did not know how to use the mask (53.0%) correctly or know which side of the FM should be on the outside (69.8%). This medium knowledge could be related to information on social media regarding the use of FMs. As mentioned by Reuter, an infographic on social media stated that the “[coloured] side should be outside if you are sick, and the white side should be outside if you are to protect yourself” [29]. This infographic is misleading and incorrect. The World Health Organization (WHO) corrected this, stating that the white side of the FM should always be inside, with the colored side outside [30]. Furthermore, according to Murphy et al., the misinformation on social media may frame a person’s knowledge of the efficacy of an intervention [31]. Thus, students should be taught how to appraise information on social media to prevent misleading concepts [32], which will affect knowledge and behavior on FM use.

This study also highlights that the majority of the students incorrectly thought that “a cloth facial mask is as effective as a regular surgical facial mask” (60.6%). This perception by health care students could be associated with misinterpretation of the information provided by the WHO that a cloth facial mask is as effective as a surgical mask provided that it is made of three layers of fabric [30]. Moreover, wearing a cloth mask is more comfortable and convenient than using a disposable FM [33]. The study also found that most students believe that a “facial mask helps to prevent human immunodeficiency virus” (71.1%). Further investigation is needed to explore the reasons for the students’ responses to this item.

Female students had higher knowledge than men. Similar findings have been observed in Saudi Arabia [17], China [19], and Vietnam [8]. A reason for this could be associated with the typical risky behavior of men regarding their health [34], which hinders them from exploring more information about FM use. This finding could also be associated with the masculine role of men in society, wherein during health threats, men must show toughness [35], which may negatively affect their interest in learning FM use.

Pharmacy students exhibited lower knowledge than nursing students. The increased knowledge of nursing students could be associated with the information that they learned in school. Nursing students are taught how to use and apply proper personal protective equipment. Part of nursing students’ training is redemonstrating the skills that they have learned, and during this process of learning, student perception is corrected. Similarly, Duong et al. reported that the training of student nurses increased their knowledge of wearing an FM [8]. These findings could be associated with the focus of courses, varied subject areas, and understanding of different colleges [18].

Regarding students’ attitudes towards FM use, the average score was 1.78 (SD = 3.99), indicating a near-neutral attitude. This result could be linked to the inconvenient experience of wearing an FM. Matusiak et al. reported that wearing FMs led to difficulty in breathing, restriction of personal freedom, inaudible speech, facial skin irritation, and sweating [36]. These inconveniences influenced a person’s attitude towards wearing an FM.

Most participants frequently wore an FM to protect themselves and prevent COVID-19. Studies in Vietnam [8] and China [16] revealed good wearing of FMs. The participants’ decision to wear FMs could be related to social and cultural practices, health-related concerns, personal meaning, and ethical and political meaning [14]. Furthermore, the practice of wearing an FM is an act of altruism and self-protection [37]. Martinelli et al. reported that the practice of an individual wearing an FM is associated with social responsibility [9]. Thus, health practitioner students wear FMs because of their social responsibility in preventing and controlling COVID-19.

In general, the findings revealed that the increased FM use KAP of health care students were associated with their perceived COVID-19-prevention knowledge. Hence, information on COVID-19 management increases students’ knowledge. The students’ knowledge of COVID-19 prevention could be due to their access to social media [30,38]. Consistent with the findings of this study, young adults in India used social media to acquire COVID-19 information such as the need to use FMs [39]. This result could be related to the perceived threat of COVID-19. Information on social media showed that the upward trend of COVID-19 cases with the new variant has had an impact on the FM use KAP of health care students. According to Arden and Chilcot, people’s change in behavior, such as using FMs, depends on their perception of the COVID-19 threat [40].

Health care students had more positive than negative attitudes towards social distancing. This finding could be associated with the perceived and actual threat of COVID-19 to health care students. Students who experienced an actual threat of COVID-19, such as knowing an individual with a confirmed case, showed more positive attitudes than those who did not know anyone infected with the virus. Feng et al. noted that a person’s knowledge of COVID-19 mitigates their behavior on social distancing [4]. The perception of the person regarding the associated risk can lead to a good attitude towards health prevention [10]. Although the decision of the person to follow social distancing policy is from an individual perspective, according to Frey et al., individual differences in the perceived risk of disease may contribute to their different responses or attitudes [41]. Risk-tolerant people are willing to take the risk [42]. Thus, this notion could be associated with the finding that health care students who have increased risk perceptions have poor social distancing attitudes.

Fourth-year and internship-year students had poorer attitudes towards social distancing than second-year students. This finding is associated with the responsibilities of those in the fourth year and internship year. The findings indicate unintentional non-adherence to social distancing due to their clinical responsibility: maintaining social distancing is impossible in the provision of care to patients.

### Limitations of the Study

The first limitation of the study is its cross-sectional method. Thus, future researchers should conduct the same study using a mixed method. Second, the study was only conducted in one university in the country; hence, the results cannot be generalized. We recommend that future research conduct a national survey of the study to generalize the finding in the Saudi context and to include the variables of pressure drops on face mask use and knowledge of the COVID-19 vaccine. Third, the tool used in the study is in English, which posed some limitations in the results because some participants may have had problems comprehending English. A psychometric study on this tool should develop an Arabic version for better comprehension by the participants. Finally, the current study examined health care students’ FM use KAP and social distancing attitudes. Consequently, future studies should examine knowledge and attitudes concerning COVID-19 vaccines among health care and nursing students.

## 5. Conclusions

This study examined the FM use KAP of health care students in Saudi Arabia as well as their attitudes towards social distancing. The findings indicate that these students had poor knowledge, neutral attitudes, and modest practices concerning the use of FM during this pandemic. Being female, being a nursing student, and having greater self-reported COVID-19-prevention knowledge were related to higher levels of FM use knowledge. Having higher FM use knowledge was related to better attitude and practice. Knowledge of COVID-19 and its prevention and decreased COVID-19 risk perception were associated with better FM use practices. Health care students had more positive than negative attitudes towards social distancing. Knowing someone who was infected by the virus, lower risk perception, and better FM use practices were associated with more positive social distancing attitudes. These conclusions have implications for health care education in Saudi Arabia and elsewhere. The findings suggest the need to ensure that future health care practitioners are knowledgeable and have positive attitudes and good practices regarding FM use as well as positive attitudes towards social distancing. These aims could be achieved by improving their FM use KAP and social distancing attitudes while they are still training for their future profession. Educational interventions should be planned and implemented among different medical-related programs and geared toward improving FM use KAP and social distancing attitudes among students. These specific preventive measures must be incorporated into the theoretical and clinical courses of these programs to avoid the knowledge–practice gap. In designing these educational interventions, the identified factors that are likely to influence the students’ FM use KAP and social distancing attitudes must be considered. For instance, an emphasis on information about FM use must be considered for pharmacy students, whereas improving nursing students’ attitudes toward FM use must be a focus. This study highlights the importance of ensuring excellent FM use KAP and social distancing attitudes among health care students because they are the future of the medical world. Furthermore, health care students must set a good example to other university students and the public about the preventive measures for COVID-19 by being knowledgeable about, having positive attitudes towards, and properly practicing these preventive measures.

## Figures and Tables

**Table 1:** Demographic characteristics of the participants (n = 543).

Variable	n	%
Sex		
Male	275	50.6
Female	268	49.4
Year		
2nd year	178	32.8
3rd year	171	31.5
4th year	157	28.9
Internship year	37	6.8
Family structure		
Nuclear family	295	54.3
Extended family	248	45.7
Program		
Nursing	298	54.9
Medicine	78	14.4
Pharmacy	118	21.7
Clinical laboratory science	49	9.0
Previous infection with COVID-19		
No	156	28.7
Yes	387	71.3
People in the immediate social environment have been infected with COVID-19		
Yes (confirmed)	295	54.3
Yes (suspected)	121	22.3
No	127	23.4
	Mean	SD
Age	22.70	4.70
Knowledge about COVID-19	6.72	2.57
Knowledge about COVID-19 prevention	7.54	2.30

**Table 2:** Results of the multiple regression analysis on the students’ face mask use knowledge (n = 543).

Predictor Variable	ß	SE-*b*	Beta	*t*	*p*	95% Confidence Interval		Collinearity Statistics	
						Lower	Upper	Tolerance	VIP
Sex	0.81	0.16	0.26	5.14	<0.001 ***	0.50	1.12	0.64	1.56
Level (Ref. group: 2nd year)									
3rd year	−0.18	0.17	−0.06	−1.08	0.281	−0.52	0.15	0.64	1.57
4th year	−0.35	0.20	−0.10	−1.74	0.083	−0.74	0.05	0.49	2.04
Internship year	−0.57	0.30	−0.09	−1.89	0.060	−1.17	0.02	0.68	1.46
Age	0.00	0.02	0.01	0.21	0.838	−0.03	0.04	0.61	1.63
Program (Ref. group: Nursing)									
Medicine	0.10	0.21	0.02	0.49	0.625	−0.31	0.52	0.74	1.35
Pharmacy	−0.36	0.18	−0.10	−1.98	0.048 *	−0.72	0.00	0.71	1.40
Clinical laboratory science	−0.35	0.26	−0.06	−1.37	0.172	−0.85	0.15	0.75	1.33
Previous infection with COVID-19	0.27	0.14	0.08	1.91	0.056	−0.01	0.55	0.96	1.04
People in the immediate social environment have been infected with COVID-19 (Ref. group: No)									
Yes (confirmed)	0.19	0.16	0.06	1.18	0.237	−0.13	0.51	0.61	1.65
Yes (suspected)	−0.08	0.20	−0.02	−0.43	0.669	−0.47	0.30	0.60	1.66
Knowledge about COVID-19	0.01	0.03	0.01	0.15	0.882	−0.06	0.07	0.62	1.63
Knowledge about COVID-19 prevention	0.09	0.04	0.13	2.46	0.014 *	0.02	0.16	0.60	1.68
Risk perception	−0.01	0.08	−0.00	−0.07	0.942	−0.17	0.16	0.68	1.47
Fear	−0.06	0.07	−0.04	−0.88	0.381	−0.19	0.07	0.78	1.28

Notes: The dependent variable was the overall mean of the face mask use knowledge. β is the unstandardized coefficient; SE-b is the standard error. *R*^2^ = 0.139; adjusted *R*^2^ = 0.114. * Significant at 0.05 level; *** significant at 0.001 level.

**Table 3:** Results of the multiple regression analysis on the students’ face mask use attitudes (n = 543).

Predictor Variable	ß	SE-*b*	Beta	*t*	*p*	95% Confidence Interval		Collinearity Statistics	
						Lower	Upper	Tolerance	VIP
Sex	0.38	0.43	0.05	0.88	0.379	−0.46	1.22	0.61	1.64
Level (Ref. group: 2nd year)									
3rd year	1.00	0.45	0.12	2.22	0.027 *	0.11	1.89	0.64	1.57
4th year	0.29	0.53	0.03	0.54	0.587	−0.75	1.33	0.49	2.06
Internship year	0.62	0.81	0.04	0.77	0.440	−0.96	2.20	0.68	1.47
Age	−0.07	0.05	−0.08	−1.42	0.156	−0.15	0.03	0.61	1.63
Program (Ref. group: Nursing)									
Medicine	1.35	0.56	0.12	2.43	0.015 *	0.26	2.44	0.74	1.35
Pharmacy	1.05	0.48	0.11	2.167	0.031 *	0.10	1.99	0.71	1.41
Clinical laboratory science	0.16	0.67	0.01	0.23	0.815	−1.17	1.48	0.75	1.33
Previous infection with COVID-19	−0.13	0.38	−0.02	−0.35	0.724	−0.88	0.61	0.96	1.05
People in the immediate social environment have been infected with COVID-19 (Ref. group: No)									
Yes (confirmed)	0.32	0.43	0.04	0.73	0.466	−0.53	1.16	0.61	1.65
Yes (suspected)	0.55	0.52	0.06	1.06	0.292	−0.47	1.56	0.60	1.66
Knowledge about COVID-19	−0.12	0.08	−0.08	−1.41	0.160	−0.28	0.05	0.62	1.63
Knowledge about COVID-19 prevention	0.05	0.10	0.03	0.55	0.581	−0.13	0.24	0.59	1.70
Risk perception	0.39	0.22	0.09	1.78	0.075	−0.04	0.81	0.68	1.47
Fear	0.32	0.17	0.09	1.83	0.068	−0.02	0.66	0.78	1.28
Face mask use knowledge	0.27	0.12	0.10	2.31	0.021 *	0.04	0.49	0.86	1.16

Notes: The dependent variable was the overall mean of the face mask use attitudes. β is the unstandardized coefficient; SE-b is the standard error. *R*^2^ = 0.073; adjusted *R*^2^ = 0.044. * Significant at 0.05 level.

**Table 4:** Results of the multiple regression analysis on the students’ face mask use practice (n = 543).

Predictor Variable	ß	SE-*b*	Beta	*t*	*p*	95% Confidence Interval		Collinearity Statistics	
						Lower	Upper	Tolerance	VIP
Sex	0.66	0.36	0.08	1.80	0.072	−0.06	1.37	0.61	1.64
Level (Ref. group: 2nd year)									
3rd year	−1.12	0.39	−0.13	−2.91	0.004 **	−1.88	−0.36	0.63	1.59
4th year	−1.60	0.45	−0.18	−3.56	<0.001 ***	−2.48	−0.72	0.49	2.06
Internship year	−2.06	0.69	−0.13	−3.01	0.003 **	−3.41	−0.72	0.68	1.47
Age	−0.04	0.04	−0.04	−0.94	0.350	−0.11	0.04	0.61	1.64
Program (Ref. group: Nursing)									
Medicine	−0.59	0.47	−0.05	−1.24	0.216	−1.52	0.34	0.73	1.37
Pharmacy	0.36	0.41	0.04	0.86	0.389	−0.45	1.16	0.70	1.43
Clinical laboratory science	−0.45	0.57	−0.03	−0.79	0.430	−1.58	0.67	0.75	1.33
Previous infection with COVID-19	−0.04	0.32	−0.01	−0.14	0.890	−0.68	0.59	0.95	1.05
People in the immediate social environment have been infected with COVID-19 (Ref. group: No)									
Yes (confirmed)	0.10	0.37	0.01	0.27	0.787	−0.62	0.82	0.61	1.65
Yes (suspected)	0.32	0.44	0.03	0.72	0.471	−0.55	1.18	0.60	1.66
Knowledge about COVID-19	0.31	0.07	0.20	4.41	<0.001 ***	0.17	0.45	0.61	1.63
Knowledge about COVID-19 prevention	0.23	0.08	0.13	2.79	0.005 **	0.07	0.38	0.59	1.70
Risk perception	−0.85	0.18	−0.20	−4.61	<0.001 ***	−1.21	−0.49	0.68	1.48
Fear	0.09	0.15	0.03	0.60	0.546	−0.20	0.38	0.78	1.29
Face mask use knowledge	0.28	0.10	0.11	2.82	0.005 **	0.08	0.47	0.85	1.17
Face mask use attitudes	−0.05	0.04	−0.06	−1.46	0.144	−0.13	0.02	0.93	1.08

Notes: The dependent variable was the overall mean of the face mask use practices. β is the unstandardized coefficient; SE-b is the standard error. *R*^2^ = 0.324; adjusted *R*^2^ = 0.302. ** significant at 0.01 level; *** significant at 0.001 level.

**Table 5:** Results of the multiple regression analysis on the students’ social distancing attitudes (n = 543).

Predictor Variable	ß	SE-*b*	Beta	*t*	*p*	95% Confidence Interval		Collinearity Statistics	
						Lower	Upper	Tolerance	VIP
Sex	0.03	0.04	0.04	0.85	0.396	−0.04	0.10	0.64	1.58
Level (Ref. group: 2nd year)									
3rd year	−0.07	0.04	−0.07	−1.71	0.088	−0.14	0.01	0.63	1.60
4th year	−0.11	0.05	−0.12	−2.45	0.014 *	−0.20	−0.02	0.48	2.10
Internship year	−0.17	0.07	−0.10	−2.51	0.012 *	−0.31	−0.04	0.67	1.49
Age	0.00	0.00	0.01	0.27	0.790	−0.01	0.01	0.61	1.63
Program (Ref. group: Nursing)									
Medicine	0.00	0.05	0.00	−0.06	0.951	−0.10	0.09	0.74	1.36
Pharmacy	0.02	0.04	0.02	0.46	0.645	−0.06	0.10	0.71	1.41
Clinical laboratory science	−0.03	0.06	−0.02	−0.56	0.575	−0.14	0.08	0.75	1.33
Previous infection with COVID-19	−0.04	0.03	−0.04	−1.19	0.233	−0.10	0.03	0.96	1.04
People in the immediate social environment have been infected with COVID-19 (Ref. group: No)									
Yes (Confirmed)	0.10	0.04	0.12	2.84	0.005 **	0.03	0.18	0.61	1.65
Yes (suspected)	0.06	0.04	0.06	1.27	0.204	−0.03	0.14	0.60	1.66
Knowledge about COVID-19	0.01	0.01	0.06	1.39	0.167	0.00	0.02	0.59	1.69
Knowledge about COVID-19 prevention	0.01	0.01	0.05	1.10	0.274	−0.01	0.03	0.59	1.71
Risk perception	−0.07	0.02	−0.16	−3.81	<0.001 ***	−0.11	−0.03	0.65	1.53
Fear	−0.02	0.02	−0.06	−1.48	0.140	−0.05	0.01	0.78	1.28
Face mask use practices	0.04	0.00	0.40	9.77	<0.001 ***	0.03	0.05	0.69	1.45

Notes: The dependent variable was the overall mean of the social distancing positive attitudes. β is the unstandardized coefficient; SE-b is the standard error. *R*^2^ = 0.400; adjusted *R*^2^ = 0.382. * Significant at 0.05 level; ** significant at 0.01 level; *** significant at 0.001 level.

## Data Availability

Data are available upon request.

## Supplementary Materials

The following supporting information can be downloaded at: https://www.mdpi.com/article/10.3390/healthcare11060901/s1, Table S1: Results of the descriptive analyses on the study variables (n = 543); Table S2: Descriptive analyses results of the risk perceptions and fear of COVID-19 (n = 543); Table S3: Descriptive analyses results on the knowledge of the correction use of face mask (n = 543); Table S4: Descriptive analyses results on the attitudes towards face mask use (n = 543); Table S5: Descriptive analyses results of the practices on the use of face masks (n = 543); Table S6: Descriptive analyses result on the attitudes toward social distancing (n = 543).
