# Supplementary material for: Face Mask Use and Social Distancing Attitude of Healthcare Students: A Multi-Disciplinary Study
Source: Healthcare (Basel). 2023 Mar 21;11(6):901. doi: 10.3390/healthcare11060901 (PMC10048679; doi:10.3390/healthcare11060901)
Supplement: Supplementary file 1 [file healthcare-11-00901-s001.zip › healthcare-2252869-supplementary.pdf]

Supplementary Table S1 Results of the descriptive analyses on the study variables (n = 543)

Variable	Mean	SD	Lowest	Highest
Risk perceptions on COVID-19	3.09	0.94	1.00	5.00
Fear of COVID-19	3.23	1.09	1.00	5.00
Face mask use knowledge	3.36	1.57	0.00	7.00
Face mask use attitudes	1.78	3.99	-11.00	11.00
Face mask use practice	7.46	3.96	0.00	12.00
Social distancing attitudes (Overall)	3.12	0.42	2.05	4.47
Social distancing positive attitudes	3.39	0.96	1.00	5.00
Social distancing negative attitudes	2.84	0.92	1.00	5.00

Supplementary Table S2 Descriptive analyses results of the risk perceptions and fear of COVID-19 (n = 543)

Risk perceptions	Very unlikely n (%)	Unlikely n (%)	Neutral n (%)	Likely n (%)	Very likely n (%)	Mean (SD)
How likely do you think it is that you might become infected with COVID-19 (Coronavirus SARS-CoV-2) in the near future?	69 (12.7)	105 (19.3)	215 (39.6)	75 (13.8)	79 (14.5)	2.98 (1.19)
How likely do you think it is that people in your family and friends might become infected with COVID-19 (Coronavirus SARS-CoV-2) in the near future?	45 (8.3)	107 (19.7)	222 (40.9)	108 (19.9)	61 (11.2)	3.06 (1.08)
How likely do you think it is to get COVID-19 (Coronavirus SARS-CoV-2) in general?	31 (5.7)	88 (16.2)	232 (42.7)	106 (19.5)	86 (15.8)	3.24 (1.08)
Fear of COVID-19	Strongly disagree n (%)	Disagree n (%)	Neutral n (%)	Agree n (%)	Strongly agree n (%)	
The COVID-19 (Coronavirus SARS-CoV-2) worries me.	50 (9.2)	77 (14.2)	172 (31.7)	146 (26.9)	98 (18.0)	3.30 (1.19)
I am afraid of being infected by COVID-19 (Coronavirus SARS-CoV-2).	64 (11.8)	89 (16.4)	170 (31.3)	133 (24.5)	87 (16.0)	3.17 (1.22)

Supplementary Table S3 Descriptive analyses results on the knowledge of the correction use of face mask (n = 543)

Variable	With correct answers n (%)	With incorrect answers n (%)
To wear a face mask correctly, the mask should cover?	255 (47.0)	288 (53.0)
What is the purpose of the metal strip on the facial mask?	372 (68.5)	171 (31.5)
Which side of the mask should face outside?	164 (30.2)	379 (69.8)
When wearing a face mask at the clinic, there is no need to cover your mouth when sneezing or coughing.	329 (60.6)	214 (39.4)
A cloth facial mask is as effective as a regular surgical facial mask.	214 (39.4)	329 (60.6)
If I am not sick, the used face mask can be stored in a bag for later use.	333 (61.3)	210 (38.7)
A facial mask helps to prevent human immunodeficiency virus.	157 (28.9)	386 (71.1)

Supplementary Table S4 Descriptive analyses results on the attitudes towards face mask use (n = 543)

Variables	Strongly disagree/ Disagree n (%)	Uncertain n (%)	Strongly agree/ Agree n (%)
Perceived susceptibility to ILI			
I am more susceptible to ILI at the clinic than in public venues	185 (34.1)	180 (33.1)	178 (32.8)
There is a high chance of having ILI transmitted to me while I am at the clinic	163 (30.0)	158 (27.1)	222 (40.9)
I feel that I am susceptible to getting ILI at the clinic	160 (29.5)	173 (31.9)	210 (38.7)
I feel that since the SARS and H1N1 crises are over, I no longer have to worry about contracting ILI	190 (35.0)	190 (35.0)	163 (30.0)
Perceived severity of ILI			
I believe that getting ILI is serious	151 (27.8)	147 (27.1)	245 (45.1)
Having ILI will be troublesome for me as I have to take time off work	151 (27.8)	181 (33.3)	211 (38.9)
I feel that if I get ILI, I will recover quickly	166 (30.6)	163 (30.0)	214 (39.4)
Having ILI will be troublesome for me as I may spread it to loved ones	135 (24.9)	141 (26.0)	267 (49.2)
Perceived benefits of wearing a face mask			
At the clinic, wearing a face mask cannot prevent the transmission of ILI.	180 (33.1)	135 (24.9)	228 (42.0)
I believe that wearing a face mask is a good way to protect myself against ILI at the clinic.	120 (22.1)	129 (23.8)	294 (54.1)
Perceived barriers to wearing a face mask			
I will only wear a face mask at the clinic if it is free	309 (56.9)	107 (19.7)	127 (23.4)
Buying a face mask at the clinic is expensive	257 (47.3)	169 (31.1)	117 (21.5)
Wearing a face mask is troublesome because I cannot communicate properly	231 (42.5)	156 (28.7)	156 (28.7)
I would feel ashamed if I was the only person wearing a face mask at the clinic	310 (57.1)	123 (22.7)	110 (20.3)
It is easier to wear a face mask if everyone at the clinic is wearing one too	176 (32.4)	122 (22.5)	245 (45.1)
Cues to action			
I would wear a face mask if there were more posters to remind me	202 (37.2)	140 (25.8)	201 (37.0)
If the doctor tells me to, I will wear a face mask	181 (33.3)	104 (19.2)	258 (47.5)
If the nurse tells me to, I will wear a face mask	142 (26.2)	125 (23.0)	276 (50.8)
Self-efficacy			
I know the proper steps for putting on a face mask	163 (30.0)	73 (13.4)	307 (56.5)

Note. ILI means Influenza-like illness

Supplementary Table S5 Descriptive analyses results of the practices on the use of face masks (n = 543)

Variable	Never/ Rarely n (%)	Sometimes n (%)	Frequently/ Always n (%)
I wear a facial mask in public venues to protect myself against ILI.	145 (26.7)	113 (20.8)	285 (52.5)
I wear a facial mask in a doctor’s clinic to protect myself against ILI.	112 (20.6)	138 (25.4)	293 (54.0)
I wear a facial mask at home when I have symptoms of ILI.	193 (35.5)	156 (28.7)	194 (35.7)
I wear a facial mask in public venues when I have symptoms of ILI.	108 (19.9)	150 (27.6)	285 (52.5)
I wear a facial mask in a doctor’s clinic when I have symptoms of ILI.	116 (21.4)	115 (21.2)	312 (57.5)
I wear a facial mask at home when family members have ILI.	147 (27.1)	149 (27.4)	247 (45.5)

Note. ILI means Influenza-like illness

Supplementary Table S6 Descriptive analyses result on the attitudes toward social distancing (n = 543)

Variable	Strongly disagree/ Disagree n (%)	Uncertain n (%)	Strongly agree/ Agree n (%)
Positive attitudes			
Social distancing has slowed the spread of coronavirus	152 (22.8)	132 (24.3)	287 (52.9)
Social distancing makes me feel safer.	137 (25.2)	123 (22.7)	283 (52.1)
It is our duty as good citizens to follow social distance orders.	137 (25.2)	123 (22.7)	283 (52.1)
For social distancing to be effective, we need everyone to follow the rules.	108 (19.9)	122 (22.5)	313 (57.6)
Stopping social distancing to soon will likely lead to another outbreak of coronavirus.	141 (26.0)	175 (32.2)	227 (41.8)
We need to have better ways to test and track people with coronavirus before we can safely lift social distance orders.	117 (21.5)	179 (33.0)	247 (45.5)
We should not lift social distance orders until there is a steady decline in the number of coronavirus cases.	121 (22.3)	160 (29.5)	262 (48.3)
We should not lift social distance orders if hospitals are full of coronavirus patients.	126 (23.2)	139 (25.6)	278 (51.2)
Negative attitudes			
Social distancing is not really doing much good.	264 (48.6)	133 (24.5)	146 (26.9)
Social distancing is doing more harm than good.	261 (48.1)	128 (23.6)	154 (28.4)
Social distance orders violate my individual rights.	222 (40.9)	173 (31.9)	148 (27.3)
Social distancing should be a matter of personal choice.	225 (41.4)	148 (27.3)	170 (31.3)
Most places in the country can safely stop social distancing.	156 (28.7)	188 (34.6)	199 (36.6)
We can lift most social distance orders and still keep coronavirus under control.	162 (29.8)	180 (33.1)	201 (37.0)
